# Engineered probiotic cocktail with two cascade metabolic *Escherichia coli* for the treatment of hyperlysinemia

**DOI:** 10.3389/fmicb.2024.1366017

**Published:** 2024-05-30

**Authors:** Feng Geng, Mingyu Wu, Pan Yang, Xueling Li, Xiaohong Pan, Yadi Wang, Junhong Lü

**Affiliations:** ^1^College of Pharmacy, Binzhou Medical University, Yantai, China; ^2^Jinan Microecological Biomedicine Shandong Laboratory, Jinan, China; ^3^College of Public Health, Shanghai University of Medicine & Health Sciences, Shanghai, China

**Keywords:** engineered probiotics, cocktail, hyperlysinemia, metabolic diseases, *Escherichia coli*

## Abstract

Engineering probiotics have emerged as a potential strategy for the treatment of metabolic diseases. However, due to the exceptional complexity of these metabolic disorders and the intricate relationship between gut microbes, it is difficult to achieve an ideal therapeutic effect in a specific metabolic disorder using only a single engineered strain. In this work, we proposed a probiotic cocktail strategy by engineering two cascade metabolic bacteria to treat hyperlysinemia, an inherited lysine metabolic disorder with loss of α-aminoadipate semialdehyde synthase (AASS) activity. A probiotic *E. coli* Nissle 1917 strain *EcNT (pTLS)* with a heterologous enzyme pathway in Saccharomyces cerevisiae was engineered to metabolize the excess lysine. Another one *EcNT (pK25)* was engineered to consume the products of lysine metabolism. The bacterial cocktail enables the maintenance of a metabolic cascade with AASS-like functional activity to maintain the blood lysine concentrations and downstream metabolites. *In vitro* experimental results showed that the cocktail bacteria had a better metabolic capacity and metabolites balance at a ratio of *EcNT (pTLS)* and *EcNT (pK25)* of 1:2. Feeding of the cocktail bacteria to the mouse model effectively reduced the concentration of lysine and balanced saccharopine in the plasma of hyperlysinemia-like mice. These findings not only provide a promising strategy of probiotic stains for the treatment of hyperlysinemia but also highlight the potential of engineered cascade cocktails to intervene and even cure other inherited metabolic diseases.

## Introduction

Engineered probiotics have emerged as novel potential therapeutic methods for the treatment of several types of human diseases, including metabolic disorders (Yadav and Shukla, 2020; Scott et al., 2021). In an inherited metabolic disorder, such as hyperlysinemia, one type of medical condition with the reduction or loss of enzyme activity caused by genetic defects interferes with the normal enzymatic metabolic processes in the body, where the accumulation or deficiency of certain metabolites gradually leads to the serious disease (Houten et al., 2013, 2014). With increasing evidence of the importance of gut microbiota in modulating host metabolic processes, probiotics have been used to regulate the production of metabolites by transforming these accumulated substances and subsequently intervening and even modifying the physiological activities in the body (Praveschotinunt et al., 2019; Zhao et al., 2022). Recently, several engineered probiotics have been developed to treat some metabolic diseases (Ma et al., 2022). For example, the engineered bacterium SYNB1618 has been successfully developed and used to treat phenylketonuria (Isabella et al., 2018; Charbonneau et al., 2020; Adolfsen et al., 2021; Puurunen et al., 2021). Given the multiple metabolites involved in the metabolic processes and the complex competition/synergy relationship between gut microbes (Cubillos-Ruiz et al., 2021; Fan and Pedersen, 2021; Lee et al., 2022), it would be interesting to test whether more than one engineered probiotic strain could achieve a better therapeutic effect via dynamically balancing the metabolites upstream and downstream in a particular metabolic disorder. In this work, we proposed a probiotic cocktail strategy by engineering two cascade metabolic bacteria to treat an inherited lysine metabolic disorder.

Hyperlysinemia is a type of amino acid metabolic disorder caused by gene mutations in α-aminoadipate semialdehyde synthase (AASS, EC 1.5.1.8/EC 1.5.1.9). In healthy human cells, AASS, together with lysine ketoglutarate reductase (LKR domain) and saccharopine dehydrogenase (SDH domain), can metabolize lysine via a two-step pathway (de Mello Serrano et al., 2012). After saccharopine is converted to α-aminoadipate semialdehyde, aldehyde dehydrogenase (ALDH) further catalyzes this to α-aminoadipate, a precursor of 2-oxoadipate acids and other metabolites. As an essential amino acid for protein synthesis, an adequate concentration of lysine is necessary to maintain a balanced metabolism. However, in patients with hyperlysinemia, mutations of either the LKR and/or SDH genes cause a reduction or loss of enzyme activity, resulting in excessive accumulation of lysine and disturbances in downstream metabolites such as saccharopine, α-aminoadipate semialdehyde, and α-aminoadipate (Sacksteder et al., 2000; Zhou et al., 2019). The inability of the intermediate metabolite might lead to many severe pathological reactions. For instance, maintaining a proper balance of α-aminoadipate concentration is crucial to neonatal neurodevelopment. A low level of α-aminoadipate may stimulate neural differentiation and facilitate ganglion cell fusion. Nevertheless, high concentrations of α-aminoadipate may result in cytotoxicity. To date, there is still no effective treatment for hyperlysinemia, and lifelong control of dietary habits is required, which is a serious life-threatening and economic burden for patients (Hoffmann and Kölker, 2012; Kou et al., 2018).

**Figure 1:**
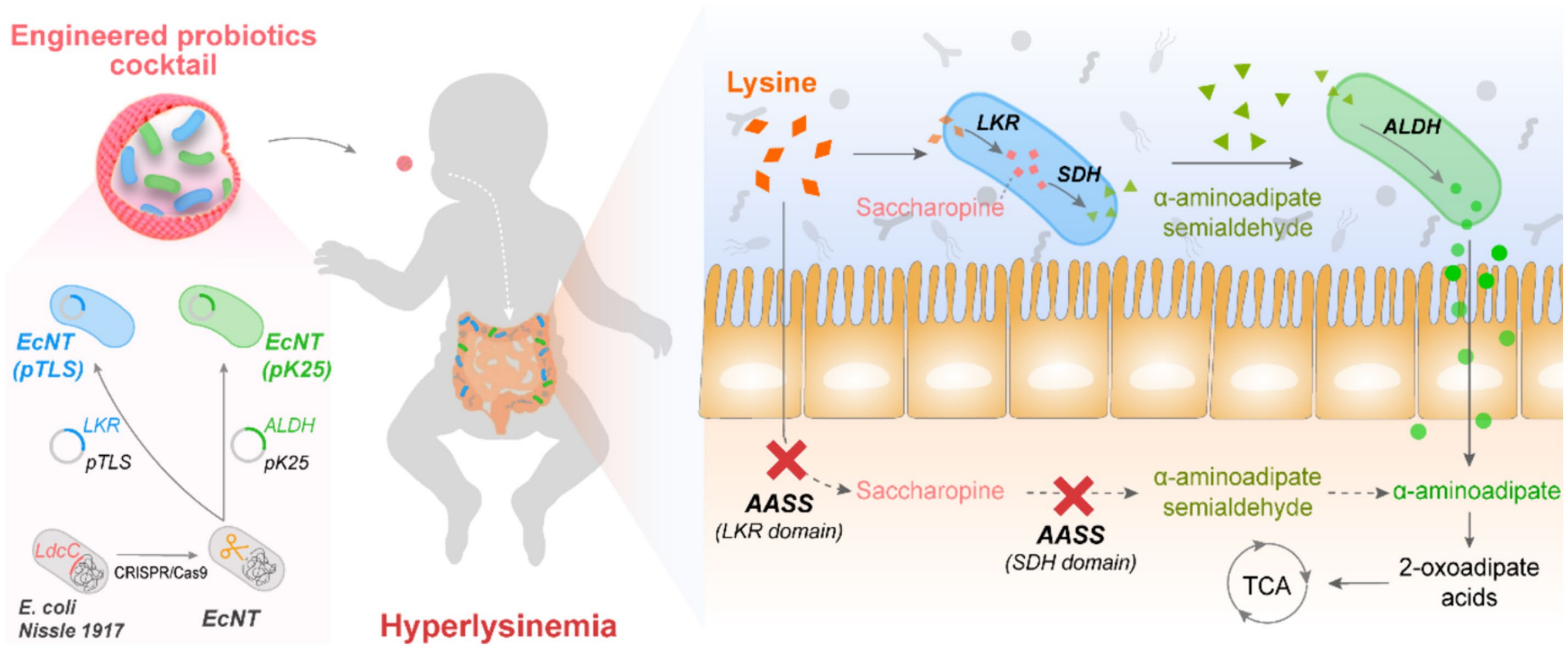
An engineered probiotic cocktail strategy with two cascade-metabolic *E. coli* strains for the treatment of hyperlysinemia Two probiotic *E. coli* strains were engineered to address the saccharopine pathway deficiency in a hyperlysinemia patient. The *E. coli* strains were designed to cascade-metabolize lysine. One strain of probiotic, *EcNT(pTLS)*, has been observed to metabolize lysine into α-aminoadipic acid semialdehyde while another, *EcNT(pK25)*, metabolizes the α-aminoadipic acid semialdehyde into α-aminoadipic acid. The enzymes involved in these processes are alpha-aminoadipate semialdehyde synthase (AASS), ketoglutarate reductase (LKR), saccharopine dehydrogenase (SDH), aminoadipate semialdehyde dehydrogenase (ALDH), tricarboxylic acid cycle (TCA).

In this work, a probiotic cocktail strategy was proposed by engineering two cascade metabolic bacteria to simultaneously replace the AASS functions in hyperlysinemia and balance the metabolite flux (Figure 1). To this end, the probiotic *E. coli Nissle 1917* (*EcN*) was selected as the host strain, and its native lysine metabolism pathway was first knocked out using CRISPR-Cas9 technology. A probiotic strain with a heterologous enzyme pathway in *Saccharomyces cerevisiae* was engineered to metabolize the excess lysine. Another one was engineered to consume the products of lysine metabolism. A cocktail of these two probiotics was constructed to obtain the optimal metabolic capacity. Finally, the possibility of using the cascade cocktail strategy to treat hyperlysinemia was tested primarily in mice.

## Materials and methods

### The construction of the engineered probiotics

The probiotic *E. coli Nissle 1917 (EcN)* is used as a host for the construction of engineered probiotics. *Lkr* and *sdh* genes were amplified from *Saccharomyces cerevisiae BY4741* and inserted into the *pTrc99a* via *Xho*I and *Nhe*I. The recombinant plasmid was designated *pTLS*. The *lys5 and lys2* genes were amplified from *Saccharomyces cerevisiae BY4741* and inserted into *pK18mobSacB* via *Xba*I and *Hind*III. The recombinant plasmid was designated *pK25*. Both recombinant plasmids were verified by sequencing. All strains were cultured in LB medium at 37°C at 200 rpm. Ampicillin (100 μg/mL), spectinomycin (50 μg/mL), kanamycin (50 μg/mL), IPTG (1 mM) or X-gal (0.1 g/L), and L-arabinose (20 mM) were supplemented as appropriate. All primers used are listed in Supplementary Table S1 in the Supporting Information.

CRISPR-Cas9 technology was used to knock out the *ldcC1* and *ldcC2* genes, which are the first enzymes in the lysine degradation pathway in *EcN*. The pGRB plasmids were used as the backbone to express sgRNA by the PCR method. The constructed gRNA plasmids were named ldcC1-gRNA and ldcC2-gRNA, respectively. The donor DNA for knocking out the *ldcC1/2* gene was obtained by three-step PCR. First, the primers ldcC1/2-up-R/ldcC1/2-up-F and ldcC1/2-down-R/ldcC1/2-down-F were used to amplify the upstream and downstream of the *ldcC1*/2 gene from the *EcN* genome. The ldcC1/2-up-R/ldcC1/2-down-R primers were then used for overlapping amplification of the two fragments to obtain ldcC1/2 donor DNA. Finally, the donor DNAs were ligated to the vectors by blunt-end ligation reactions and verified by sequencing, respectively. The *ldcC1* and *ldcC2* knockout genes were applied according to Li et al. (2015). The *EcN(pREDCas9)* competent cells were generated by electroporation. The donor DNA and gRNA plasmid were transformed into *EcN(pREDCas9)*. Clones were identified by colony PCR and DNA sequencing. The correct clone was inoculated into LB with spectinomycin and 10% L-arabinose and cultured overnight to lose the gRNA plasmids. Finally, the *EcN* of the *ldcC1* gene knockout was named *EcN ∆ldcC1 (EcNO)*. The *EcNO* was used in the next round for *ldcC2* gene editing to obtain *EcN ∆ldcC1 ∆ldcC2* (*EcNT*). The recombinant plasmids were transformed into *EcNT* and the recombinant strains were named *EcNT (pTLS)* and *EcNT (pK25)*. The primers used to amplify the pGRB plasmids or donor DNA are listed in Supplementary Table S2.

### The cultures of the engineered strains in shaken flasks

The metabolic characteristics of *EcNT (pTLS)* and *EcNT (pK25)* were investigated in shaken flask cultures. The engineered *EcN*s were cultured as seeds in LB medium overnight. The seeds were then cultured at 37°C in the fermentation medium (10 g/L glucose, 1 g/L yeast extract, 5 g/L KH_2_PO_4_, 5 g/L (NH_4_)_2_SO_4_, 0.7 g/L KCl, 0.003 g/L FeSO_4_ ·7H_2_O, 0.003 g/L MnSO_4_, 1 g/L MgSO_4_). Cultures were grown at 37°C with an initial OD_600_ of 0.1 and 0.1 mM isopropyl-B-D-thiogalactoside (IPTG). Samples were taken at regular intervals for measurement of cell growth (OD_600_), sugar concentration, and metabolite analysis. All fermentations were performed with three samples in parallel. Glucose concentration was determined using a glucose assay kit with the glucose oxidase method. Lysine concentration was determined by high-performance liquid chromatography (HPLC LC-2030). Sample pretreatment was performed according to the Dikma protocol of using Diamonsil AAA 5 μm (4.6 mm × 250) column and ultraviolet detector at 360 nm. Mobile phase A was 0.02 mol/ L Na_2_HPO_4_ and 0.02 mol/ L NaH_2_PO_4_ aqueous solution, mobile phase B was methanol: acetonitrile =10:90 (V: V). The column temperature was maintained at 45°C and the flow rate was 1 mL/min. The analysis was performed using a binary gradient elution method according to the protocol of Dikma. Growth rates were calculated using the method (OD_6h_-OD_0h_)/OD_0h_; rates of glucose as well as lysine metabolism were calculated using the method (C_6h_-C_0h_)/C_0h_/OD.

### Preparation of microsphere system with the engineered strains

The *EcNT* strains were inoculated into LB medium, and cultured overnight at 37°C and 200 rpm for seeds. Seeds were inoculated into the fermentation medium as above with the addition of 10 g/L lysine and cultured at 37°C 200 rpm for 6 h. The activated bacterial solution was collected in a sterile 2 mL centrifuge tube, resuspended with deionized water to wash 3 times, and incubated at 10^8^ CFU/mL. 2% sodium alginate microbial colloid was dropped into a 4% CaCl_2_ aqueous solution at a uniform rate and stirred on a magnetic stirrer. The microsphere was centrifuged for 10 min at 4°C at 5000 rpm, washed three times with sterile water to remove CaCl_2_, and resuspended in sterile water. The microspheres are poured into the sterile medium and frozen in a lyophiliser for 12 h. After spraying with gold, the microspheres were photographed using an electron microscope. The microspheres were washed three times with sterilized water and cultured in 20 mL of fermentation medium at 37°C 150 rpm for 24 h for the stability test. Cell growth (OD600) and sugar concentration were tested as above. All samples were tested in triplicate.

### The verification of the engineered cascade cocktail in mice

To verify the cascade cocktail of engineered strains in mice, C57BL/6 mice (age, 6 weeks; weight, 18–20 g; half male and half female) were purchased from the Jinan Pengyue Experimental Animal Center (China) and fed under specific pathogen-free (SPF) conditions at the Experimental Animal Center of Binzhou Medical University. The experiment was conducted after 2 weeks of adaptive feeding. Male and female mice were divided into 4 cages with 5 mice in each cage. The mice were randomly assigned to control, *EcN*, *EcNT*, *EcNT*(*pTLS*), *EcNT*(*pK25*), and the probiotic cocktail of *EcNT*(*pTLS*) and *EcNT*(*pK25*) in a ratio of 1:2, with 10 mice in each group. The probiotic solution was mixed with the strains and inoculated into 50 mL of LB liquid medium at OD = 0.1 and cultured overnight at 37°C and 200 rpm. The next day, the cells were collected by centrifugation at 5000 rpm for 10 min, washed twice with sterilized ultrapure water, and resuspended in sterilized water to prepare a bacterial solution for probiotic administration. The probiotic groups were alternately fed with lysine solution for 1 day and the probiotic solutions for the next, while the control group was fed with lysine solution for 1 day and PBS solution for 1 day during the experiment. To maintain the activity of the probiotics, the fresh liquid of the probiotics was changed every feeding day. The body weight of the mice was recorded from the first day of administration and then every 5 days/time until the end of the experiment. Mice were sacrificed at 12 weeks of age after 6 weeks of drug-feeding. Three mice in the drug group and control groups were randomly selected and faecal; and plasma samples were collected. Fecal samples were collected with sterile forceps and 3–5 pieces of fresh mouse feces were placed in sterile cryopreservation tubes, immediately frozen with liquid nitrogen for 30 min, and then stored at −80°C. Blood was collected in heparin-sodium anticoagulant tubes at 5000 rpm for 5 min, and plasma was collected. The plasma sample was pretreated as follows: the sample was placed into a 1 mL centrifuge tube and the same volume of methanol was added for protein precipitation, centrifuged at 15,000 rpm after sonication for 2 min, the supernatant was taken and passed through a C_18_-containing membrane and placed it in a sample bottle. The samples were analyzed by HPLC (SHIMADZU-20A) and Tandem mass spectrometry (MS/MS) (Applied Biosystems 5,500 Quadrupole Trap) using WAX column (5 cm × 4.0 mm) at 45°C and a flow rate of 0.5 mL/min. The mobile phase A was 5 mmol/L ammonium acetate 0.1% formic acid in water and B was methanol. The elution gradient was 0–2.0 min, *B* = 90%; 2.1–4.0 min, *B* = 70%; 4.1–6.0 min, *B* = 50%; 6.0–8.0 min, *B* = 30%; 8.1–10 min, *B* = 10%; 10.1–13.0 min, *B* = 90%. The mass spectrometry ion source was ESI. The condition was curtain gas (psi): 38; ionization voltage (V): positive ion 5,500; temperature (°C): 550; spray gas (psi): 55; auxiliary heating gas (psi): 55; scan mode: MRM.

The experimental data were processed and analyzed using Prism 9. The lysine metabolic efficiency was calculated following lysine metabolic efficiency = (1- Conc. lysine in treated group/ Conc. lysine in EcNT) *100%. Relative saccharopine concentrations were calculated following Relative Conc. saccharopine = Conc. saccharopine in treated group/ Conc. saccharopine in EcNT *100%. Error bars represent the coefficient of variation. All data analyses were considered statistically significant at *p* < 0.05. Gut microbial diversity was tested at Shanghai Qrigingene Biotechnology Co., Ltd. for 16 s RNA sequencing analysis. The Illumina PE250 high-throughput sequencing platform was used for sequencing. Animal protocols were approved by the Ethics committee of the Binzhou Medical University No. 2021049 and all experiments were conducted following the National Guidelines for the Ethical Review of Laboratory Animal Welfare of the People’s Republic of China (GB/T 35892–2018).

## Results

### An engineered probiotic cocktail strategy with cascading lysine metabolism for treating hyperlysinemia

Hyperlysinemia, which involves high levels of amino acid lysine in the blood, is caused by the absence of the AASS enzyme. Our proposal involves introducing gut bacteria similar in activity to the AASS enzyme to improve the breakdown of lysine in the blood and urine. To investigate this hypothesis, we formulated a probiotic mixture that would metabolize lysine and its downstream metabolites sequentially (Figure 1). Since the inherent lysine metabolism in probiotic *E. coli Nissle 1917* (*EcN*) is mediated by the cadaverine pathway, whose metabolites are highly toxic to the host cells, we initially modified the host *EcN* strain by using CRISPR-Cas9 technology to eliminate its *LdcC1* and *LdcC2* genes. Subsequently, we developed two probiotic strains that can carry out lysine metabolism with the saccharopine pathway. Given that the expression of the human-derived AASS protein in *E. coli* is unstable, whereas yeast has the same lysine degradation pathway as humans with better solubility and stability in bacteria, two enzymes involved in yeast lysine metabolism, lysine-ketoglutarate reductase (EC1.5.1.7, abbreviated as LKR, encoded by the *lys1* gene) and saccharopine dehydrogenase (EC1.5.1.10, SDH, encoded by the *lys9* gene) were selected for the production of the engineered probiotic bacteria. One *EcN* strain has been engineered to convent lysine to α-aminoadipic acid semialdehyde by heterologous overexpression of LKR and SDH from *Saccharomyces cerevisiae*. The alternative *EcN* strain has been genetically modified to convent α-aminoadipic acid semialdehyde to α-aminoadipic acid by overexpression of *Saccharomyces cerevisiae*’s aminoadipate semialdehyde dehydrogenase (EC1.2.1.3, ALDH). Finally, an engineered probiotic cocktail consisting of two strains was used to achieve balanced lysine metabolism.

### The construction and verification of engineered probiotics

**Figure 2:**
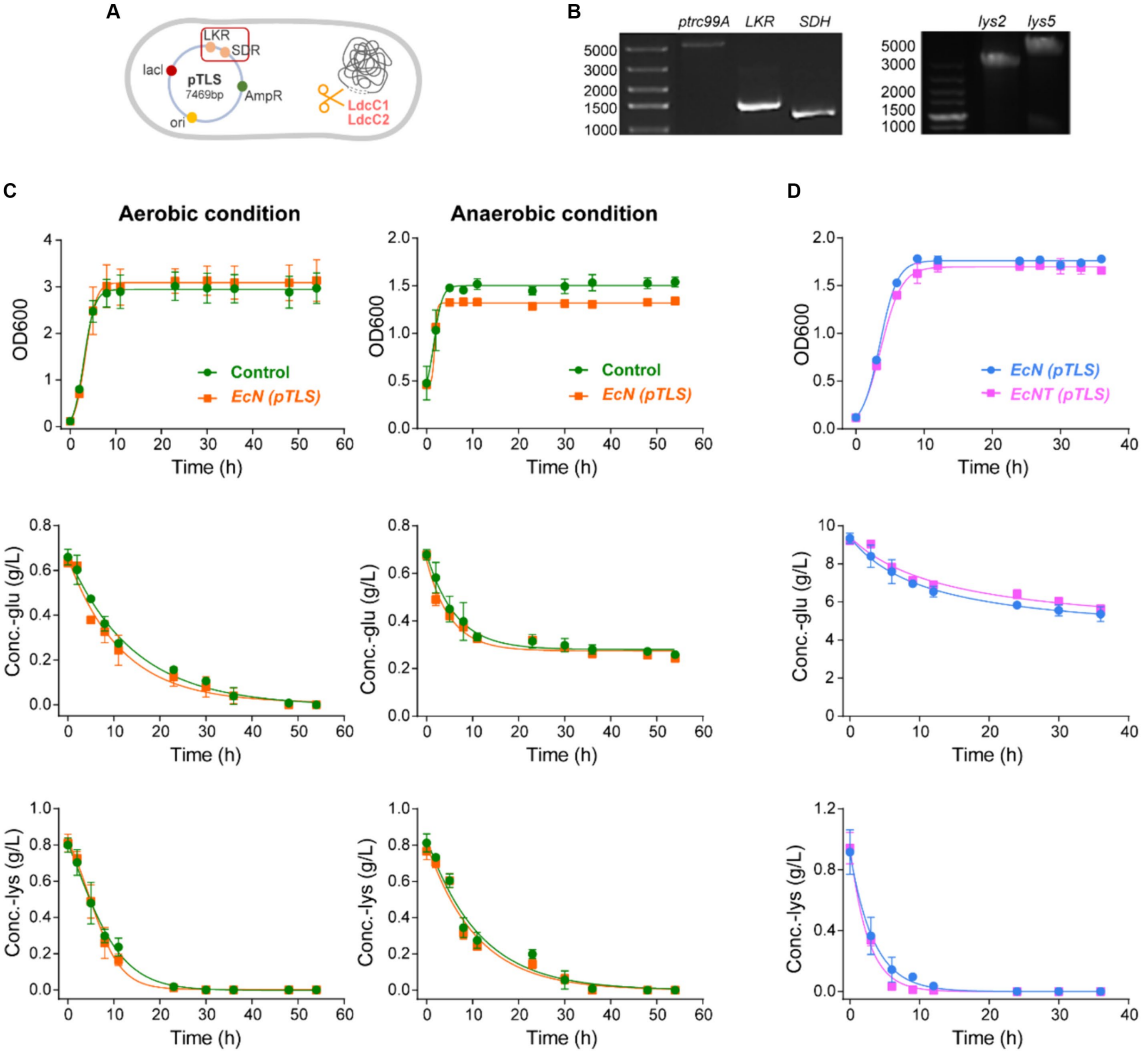
The construction and verification of engineered probiotics. **(A)** *EcNT (pTLS)* were designed to create an engineered probiotic cocktail from the *EcN*. CRISPR-Cas9 technology was employed to construct *EcNT* by knocking out the *ldcC* genes responsible for lysine degradation. *EcNT (pTLS)*, which expressed plasmid *pTLS*, was used to constitutively express ketoglutarate reductase (LKR) and saccharopine dehydrogenase (SDH) from *Saccharomyces cerevisiae BY 4741*. **(B)** The colony PCR of the plasmids. The left figure is the colony PCR of the plasmid *pTrc99a* and genes LKR and SDH. The right figure is the colony PCR of *pTrc99a -lys2* and *pTrc99a-lys5*. The *lys2* and *lys5* genes are cloned from *Saccharomyces cerevisiae BY 4741*. We clarify this in the figure caption. **(C)** The growth and metabolic capacity of the *EcNT (pTLS) and EcNT (ptrc99A)* were analyzed under both aerobic and anaerobic conditions. **(D)** The growth and metabolism of wild-type *EcN (pTLS)* strain and engineered *EcNT (pTLS)* train with plasmid *pTLS*.

We chose the probiotic *EcN* as the host to create an engineered probiotic cocktail utilizing two cascade-metabolic *E. coli*. Initially, two recombinant plasmids were developed to overexpress the AASS-like enzymes in the saccharopine pathway. A plasmid called *pTLS* was constructed by utilizing *pTrc99a* and expressing ketoglutarate reductase (LKR) and saccharopine dehydrogenase (SDH) from *Saccharomyces cerevisiae BY4741* (Figures 2A,B). This plasmid was able to convert lysine into saccharopine through to saccharopine semialdehyde. The additional plasmid, *pK25*, was produced from *pK18mob* featuring kanamycin resistance and a perpetually expressed aminoadipate semialdehyde dehydrogenase (ALDH, encoded by *lys2* and *lys5*). This facilitated the conversion of saccharopine semialdehyde to α-aminoadipic acid. Both plasmids metabolize excess lysine to α-aminoadipic acid, which is degraded via the tricarboxylic acid (TCA) cycle. Unlike mammalian cells, the lysine catabolism pathway in *E. coli* cells produces harmful cadaverine. To address this, we used CRISPR-Cas9 technology to remove the *ldcC* genes responsible for breaking down lysine in *EcN*. The engineered *EcN* strain was then designated as *EcNT*. Subsequently, we introduced the *pTLS* and *pK25* plasmids into *EcNT*, producing two recombinant strains named *EcNT (pTLS)* and *EcNT (pK25)*, respectively. These two engineered bacteria collaborate to form a cascade. *EcNT (pTLS)* converts lysine to α-aminoadipate semialdehyde, followed by the conversion of α-aminoadipate semialdehyde to non-toxic alpha-aminoadipic acid by *EcNT (pK25)*. This combination of strains achieves the dual purposes of providing supplements for pathogenic AASS and balancing the dynamic cascade metabolites from lysine simultaneously.

Shaken flask experiments were conducted to explore the metabolic traits of the engineered bacteria *in vitro*. Initially, these bacteria must endure the anaerobic gut environment to attain the curative objective. Although the wild-type *E. coli* is a facultative anaerobium capable of survival in anaerobic intestinal conditions, the competence of the engineered derivative in performing within the gut environment necessitates authentication. The engineered strains’ metabolic capacity and growth were examined with and without oxygen, in which shaken flask experiments illustrated that *EcN (pTrc99a)* and *EcN (pTLS)* had comparable growth rates under both aerobic and anaerobic conditions (Figure 2C). The engineered modifications did not impede *EcN*’s oxygen tolerance, i.e., the engineered EcNs can thrive and metabolize in the presence or absence of oxygen. Furthermore, the *ldcC1* and *ldcC2* genes within *EcN*’s genetic makeup are responsible for the catabolism of lysine into cadaverine, which is both highly toxic and irritating. Consequently, we utilized CRISPR-Cas9 technology to remove *ldcC1* and *ldcC2* genes from the wild-type strain of *EcN*, resulting in an engineered strain named *EcNT*. To assess the differences in growth and metabolism between the wild-type *EcN* strain and the engineered *EcNT* strain with plasmid *pTLS*, we carried out a shake-flask experiment. The findings revealed that both the modified strain and wild-type strain exhibited similar rates of growth and sugar consumption. However, the *EcNT(pTLS)* strain depleted lysine earlier than the *EcN(pTLS)* strain (Figure 2D). Hence, the genetic modifications made have had no significant impact on the *EcN* strain’s growth and metabolism. Additionally, the recombinant plasmid exhibits consistent expression.

### Optimization of the cascade cocktail by two engineered *EcN*s *in vitro* by an artificial microsphere system

**Figure 3:**
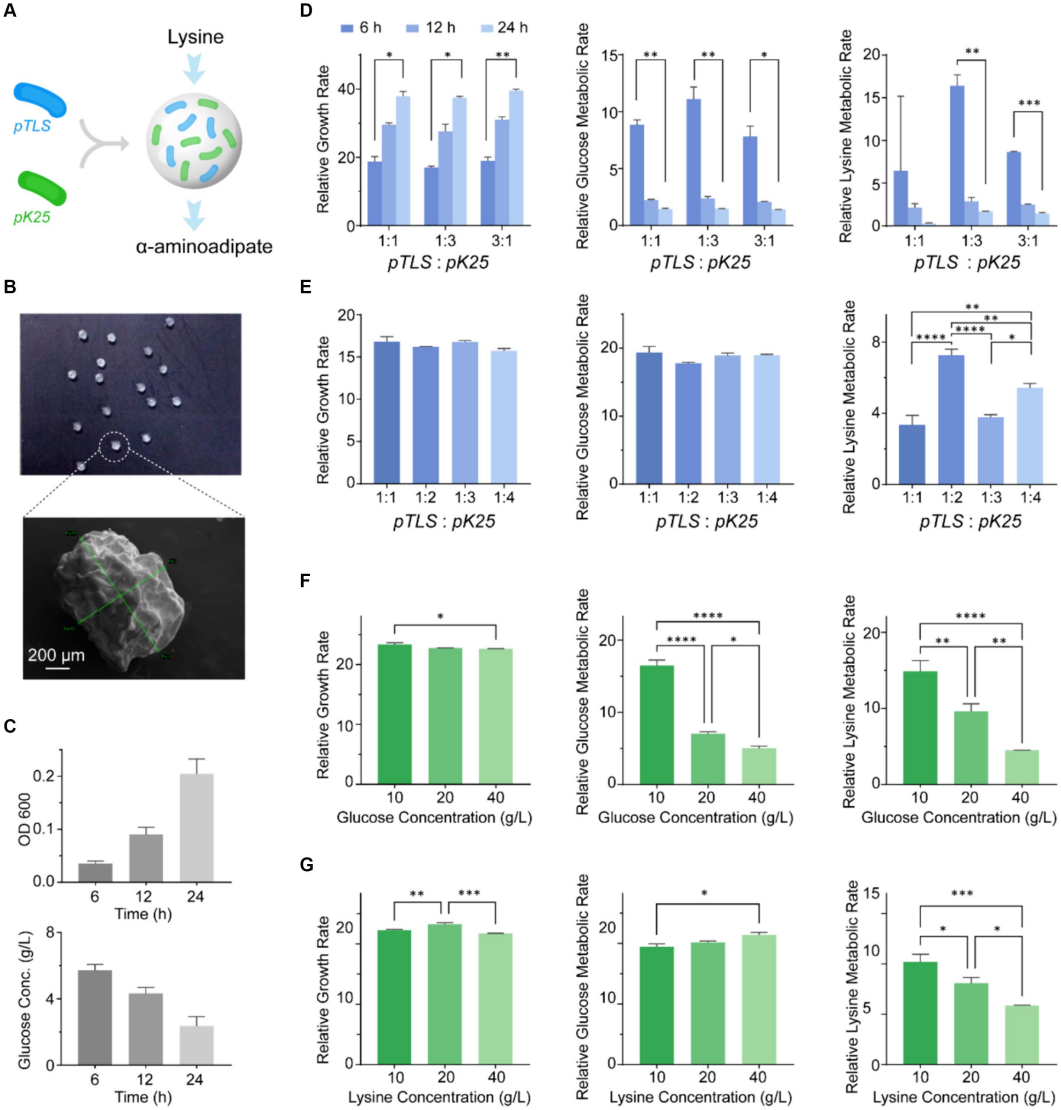
The *in vitro* optimization of the cascade cocktail with two engineered *EcN*s and the construction of an artificial microsphere system. **(A)** A microsphere system was designed to enable lysine metabolism between different bacterial strains through a metabolic cascade, achieved by co-localizing *EcNT (pTLS)* and *EcNT (pK25)*. **(B)** The morphology of the microspheres composed of *EcNT (pTLS)* and *EcNT (pK25)* with a ratio of 1:2 coated with sodium alginate. **(C)** The fermentation process was used to evaluate the dissolution and metabolism of the microspheres. **(D)** The growth rate, glucose consumption, and lysine metabolic rate of the synthetic microbiome for different *EcNT (pTLS)* and *EcNT (pK25)* ratios (1:1, 1:3, and 3:1). **(E)** The growth rate, glucose consumption, and lysine metabolism of *EcNT (pTLS)* and *EcNT (pK25)* for ratios of 1:1, 1:2, 1:3, and 1:4. **(F,G)** The probiotic cocktail’s tolerance to glucose **(F)** and lysine **(G)** in the medium with a 1:1 bacteria ratio. Glucose or lysine concentrations of 10 g/L, 20 g/L, and 40 g/L were used.

The engineered bacteria must cooperate in the complex gut environment to achieve therapeutic objectives. However, various bacteria colonize different locations within the gut, where spatial partitions can interrupt material exchange between the two bacteria. Consequently, we investigated an artificial microsphere system to encourage the metabolic cascade reactions between different strains that have spatial colocalizations. We refined the metabolic properties of the cascade cocktail and established an artificial microsphere system comprising *EcNT (pTLS)*, herein referred to as *pTLS*, and *EcNT (pK25)*, also known as *pK25* (Figure 3A). Bacteria cultured for 6 h at a 1:2 ratio were coated with sodium alginate to produce microspheres (Figure 3B). Microsphere morphology was examined using a scanning electron microscope. The microspheres are approximately 1 mm in diameter and have a rough surface. Microsphere dissolution and metabolism were evaluated via fermentation (Figure 3C). The study demonstrated that the microbial microspheres remained intact and there was minimal bacterial liquid dissolution in the medium within 24 h, thus indicating the stability and effectiveness of the artificial ecosystem microspheres.

To investigate the growth and metabolic capacity of the engineered strains, we initially analyzed the ratio of bacteria and the concentrations of medium components *in vitro*. We investigated the prevalent strains of two different bacteria in a cascade cocktail and analyzed their impact on growth, sugar consumption, and lysine metabolism. To identify the dominant strain, we established the ratio of *EcNT (pTLS)* and *EcNT (pK25)* was set as 1:1, 1:3, and 3:1 while monitoring the growth rate of the engineered strains under varying ratios of glucose, and lysine. We measured the metabolic rates at 6, 12, and 24 h to determine their effects. Figure 3D illustrates that engineered strains with differing dominant strains exhibited comparable growth rates over the same period. However, the metabolic rate at 6 h was significantly higher than that of the other two periods. Notably, the engineered microbiome displayed a higher level of lysine metabolism compared to the other two groups when *EcNT (pK25)* was the dominant bacterium. Therefore, the engineered strains with *EcNT (pK25)* as the dominant bacterium demonstrated faster lysine metabolism. We conducted a comparison of the ratios of *EcNT (pTLS)* and *EcNT (pK25)* at 1:1, 1:2, 1:3, and 1:4 to examine the effect of *EcNT (pK25)* on lysine metabolism in the cocktail. Figure 3E displays that the growth and glucose metabolic rates of each system were comparable, while the lysine metabolic rate at a 1:2 ratio of *pTLS:pK25* was greater than the others. Moreover, we compared the tolerance of the engineered strains to glucose and lysine in the medium at a 1:1 ratio. Glucose concentrations of 10 g/L, 20 g/L, and 40 g/L were compared in shake flasks. The results are demonstrated in Figure 3F, indicating that high initial glucose concentration impeded both glucose and lysine metabolism. Furthermore, the outcome of lysine concentrations of 10 g/L, 20 g/L, and 40 g/L as the initial medium components (Figure 3G) reveals that the high lysine concentration decreased the speed of lysine metabolism.

Overall, the two-strain cascade cocktail system was affirmed through *in vitro* testing, with the highest metabolic capacity found in *EcNT (pTLS)*: *EcNT (pK25)* at a 1:2 ratio. Additionally, an artificial microsphere system was created to address the issue of spatial colocalizations.

### The validation of the cascade cocktail by two engineered probiotics in mice

**Figure 4:**
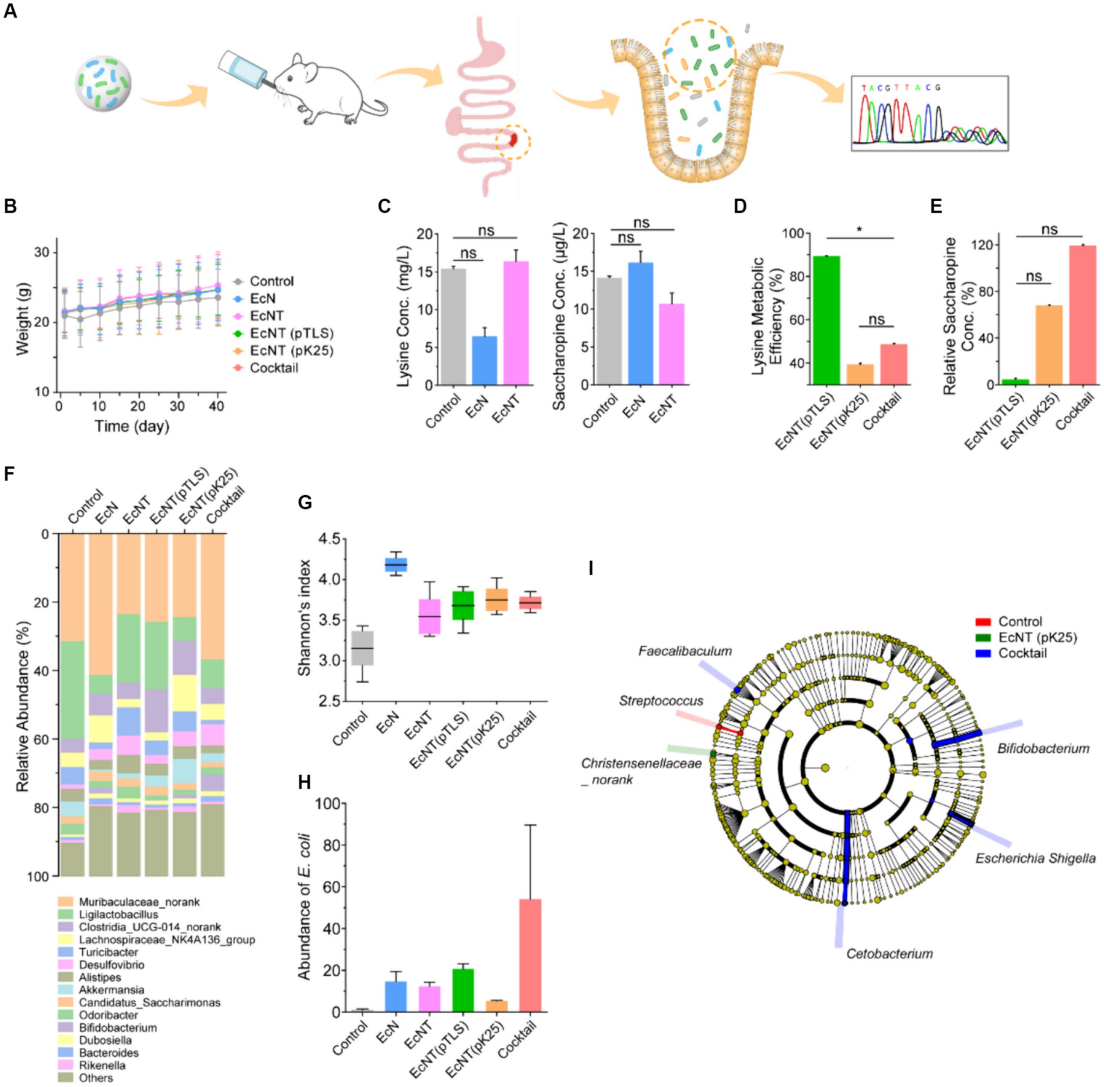
*In vivo* therapeutic effect of engineered probiotics in mice. **(A)** The engineered probiotics were administered to hyperlysinemia-like mice to determine their effect on hyperlysinemia. The C57BL/6 mice were reared on a high level of lysine diet (40 g/L) to simulate the high lysine blood by hyperlysinemia. The hyperlysinemia-like mice were divided into water, *EcN*, *EcNT*, *EcNT*(*pTLS*), *EcNT*(*pK25*), and the probiotic cocktail of *EcNT*(*pTLS*) and *EcNT*(*pK25*) in a 1:2 ratio. Each group was fed with water or strains for 6 weeks. Blood samples were taken after 6 weeks for biological evaluation. The status of the gut microbiome in mice of control and cocktail mice was measured by 16S rRNA sequencing after administration. **(B)** Weight changes of the mice during the treatment period. **(C)** Analysis of lysine and saccharopine concentrations in the plasma of mice fed with water, *EcN*, *and EcNT* by *LC–MS/MS*. The serum lysine concentration of control, *EcN, EcNT, EcNT(pTLS*), *EcNT(pK25)*, and the cocktail are 15.4 ± 0.3, 6.5 ± 1.2, 16.4 ± 1.5, 1.73 ± 0.9, 10.0 ± 0.4, and 8.4 ± 0.4 and serum saccharopine concentration of control, *EcN*, *EcNT*, *EcNT(pTLS) EcNT(pK25)*, and the cocktail are *14.1 ± 0.3*, *16.2 ± 1.5*, *10.7 ± 1.4*, *0.5 ± 1.0*, *7.3 ± 0.6 and 12.8 ± 0.9*, respectively. **(D)** Analysis of the lysine metabolic efficiency of *EcNT(pTLS), EcNT(pK25)*, and the cocktail compared to *EcNT*. The lysine metabolic efficiency was calculated as *lysine metabolic efficiency =* (1- Conc. lysine in treated group/ Conc. lysine in EcNT) *100%. **(E)** Relative plasma saccharopine concentrations in the plasma of mice of *EcNT*(*pTLS*), *EcNT*(*pK25)*, and the cocktail compared to *EcNT*. Relative saccharopin concentrations were calculated as Relative Saccharopine concentration = Saccharopine concentration in treated group/ Saccharopine concentration in EcNT *100%. **(F)** The microbial community bar plot. **(G)** The Shannon index. **(H)** The abundance of *E. coli*. **(I)** The differential analysis of the cladogram plot. The error bars in panels **(C–E)** represent the coefficient of variation, while the other figures show SD.

Next, we examined the effect of the probiotics on a hyperlysinemia-like mice model. C57BL/6 mice were raised on a lysine diet of 40 g/L to obtain high blood lysine concentrations mimicking the hyperlysinemia condition (Figure 4A). To evaluate the mice’s blood lysine sensitivity, they were split into three groups and fed with water, *EcN*, or *EcNT* for 6 weeks, respectively. During the 40-day treatment period, there were no noteworthy differences observed among the various groups (Figure 4B). Following the treatment, the plasma of the mice was examined to evaluate the concentrations of lysine and saccharopine via *LC–MS/MS*. As depicted in Figure 4C, in comparison with the control group, the *EcN* group was able to decrease lysine with a significant accumulation of saccharopine. It is plausible that the decreased lysine was metabolized using the cadaveramines pathway from *E. coli*. This can be demonstrated by the elevated blood concentration of lysine level in the *EcNT* group with the deleted *ldcC1* and *ldcC2* genes that regulate *EcNT*’s cadaverine pathway. Furthermore, the findings indicate that the hyperlysinemia-like mice model is responsive to the assessment of blood lysine levels.

We further conducted research on probiotics in mice with hyperlysinemia-like symptoms to determine their impact on hyperlysinemia. The hyperlysinemia-like mice were administered with *EcNT(pTLS)*, *EcNT(pK25)*, and a mix of probiotics, *EcNT(pTLS*) and *EcNT(pK25)* at a ratio of 1:2 for 6 weeks. During the 40-day treatment, we observed that the weight changes in the treatment group were not significantly different (Figure 4B) compared to the control group. The mouse plasma was analyzed using LC–MS/MS after treatment. The *EcNT(pTLS)* treatment efficiently lowered the levels of plasma lysine and saccharopine, as anticipated. The metabolic rates of lysine and saccharopine were higher in the *EcNT(pTLS)* group by roughly 89 and 95%, respectively when compared to the *EcNT* group (Figures 4D,E). The rapid rates at which lysine is metabolized by *EcNT(pTLS)* suggest that the synthetic bacteria’s lysine degradation pathway can effectively process free lysine *in vivo*. Furthermore, *EcNT(pTLS)* could lower the quantity of lysine and saccharopine in plasma immediately after protein digestion.

A suitable concentration of lysine is essential for protein synthesis, and it is necessary to maintain a balanced metabolism. Excessive consumption of lysine and saccharopine can hinder metabolism maintenance. The cocktail probiotics of *EcNT(pTLS)* and *EcNT(pK25)* reduce the lysine concentration by 45% and balance saccharopine concentration, which is more appropriate for balancing the lysine concentration required for protein synthesis and the downstream metabolites compared to *EcNT(pTLS)*. Furthermore, the cocktail group could balance the downstream metabolites through large fluxes of lysine metabolism. To sum up, the cascade cocktail of engineered probiotics could effectively decrease lysine content, maintain saccharopine concentration equilibrium in plasma, and potentially mitigate the side effects of lysine’s incomplete metabolism in hyperlysinemia-like mice.

To compare the gut microbiota of mice with and without the different strains, we conducted 16S rRNA sequencing to detect changes in the gut microbiome. The results of the Shannon analysis showed that mice administered with *EcN* exhibited a significant increase in microbiome diversity. Additionally, mice administered with the engineered strains, namely *EcNT*, *EcNT(pTLS)*, *EcNT(pK25)*, and the cocktail, also showed increased microbiome diversity compared to the control group (Figure 4G). Diversity analysis indicated that the cocktail treatment rendered the composition of the microbiome similar to that of the *EcN* group, suggesting a positive effect on the gut’s healthy microbiome (Figure 4F). Various taxa of commensal bacteria were found to increase in number in the cocktail group as observed in the taxonomic composition of microorganisms, including bacteria belonging to the genus *E. coli* (Figure 4H). We also found that *Bifidobacterium*, *Escherichia*, *Cetobacterium*, and *Faecalibaculum* were significantly increased in the cocktail group (Figure 4I). In addition, *Streptococcus* was significantly reduced in the control group and *Christensenllaceae* was significantly reduced in the single-engineered strain group. (Figure 4I). These results demonstrate that the engineered strains can reach the intestine of mice and that the engineered cocktail slightly affects the composition of the gut microbiota without inducing microbiome dysregulation.

## Discussion

The cascade cocktail composed of more than one engineered probiotic has several advantages. First, complex metabolic diseases such as hyperlysinemia are often caused by multiple genetic mutations and require multiple metabolic enzymes in the complementary pathway to maintain metabolic balance. Such diseases involve disorders of multiple metabolites that are difficult to treat with conventional drugs. We have presented cascade multi-bacterial therapy as a novel idea for the treatment of multi-metabolite disorders. Different engineered bacteria work together to metabolize lysine and its related metabolites through cascade metabolism, which can significantly alleviate the adverse effects caused by metabolic disorders. Second, metabolomics showed that the supplementation of multi-engineered bacteria constructed with *E. coli* could colonize the intestine and help metabolize lysine to reduce blood concentrations of lysine and saccharopine, which achieved long-term treatment of metabolic diseases without disturbing the intestinal ecosystem. In addition, the spatial colonization of engineered bacteria could be realized by coating, which would maintain the bacterial ratio to achieve efficient and rapid metabolism and reduce the excretion and loss of intermediate metabolites (Zheng et al., 2020). The multi-bacterial strategy would be expected to achieve precise regulation of abnormal metabolites in the gut, which could be widely used in the personalized and adjuvant therapy of complex diseases.

A suitable concentration of lysine is essential for protein synthesis, and it is necessary to maintain a balanced metabolism. The role of the first strain is to degrade lysine to produce the intermediate. However, excessive consumption of lysine and saccharopine can hinder metabolism maintenance. A certain concentration of the lysine metabolic intermediates, such as aminoadipate, is also required by the body. The second strain degrades part of the intermediate, allowing the metabolic flow to proceed smoothly. Therefore, the cascade cocktail of engineered probiotics could effectively decrease lysine content, maintain saccharopine concentration equilibrium in plasma, and potentially mitigate the side effects of lysine’s incomplete metabolism in hyperlysinemia-like mice. Here we first prove of concept to validate the therapeutic effect of engineering bacteria based on normal mice with high levels of blood lysine concentration. In our experiment, mice fed with high lysine concentration water were able to maintain high lysine levels in their blood, which proves that lysine in the diet can enter the bloodstream and affect the concentration of lysine in the blood. Moreover, mice treated with engineered bacteria can reduce blood lysine levels, which proves of concept to validate the therapeutic effect of engineering bacteria to degrade lysine *in vivo*. The model mice in this study (Zhou et al., 2019) will be used in future experiments to validate the therapeutic effect of engineering bacteria in the treatment of AASS mutant hyperlysinemia.

We have verified the potential of engineered cocktail therapy in the treatment of hyperlysinemia, while the system can also be improved in the following aspects. First, plasmids were used to express heterogeneous genes in the current engineered bacteria. Although no significant plasmid loss was observed in the experiments, it is better to integrate the genes into the genome of the engineering bacteria using gene/genome editing technology to fundamentally solve the problem of plasmid stability and avoid the effect of resistance genes. The expression level of genes can be also optimized by promoter engineering. Second, this study explored the possibility of the microsphere system with mixed probiotics *in vitro*. As the size of the constructed microspheres was not suitable for administration by gavage needle, we only verified the mixed bacteria in the mouse experiment. A microsphere system that is more suitable for oral administration will be developed. In addition, pseudo-high lysine mice were used to study the therapeutic effect of the engineered bacterial cocktail on hyperlysinemia. Recently, the hyperlysinemia model mice were developed by gene knockout (Zhou et al., 2019) In the future, gene knockout hyperlysinemia model mice may be used to improve the cascade cocktail of engineered strains.

## Conclusion

In this work, we proposed a cascade cocktail strategy with two engineered probiotics for the treatment of hyperlysinemia. We demonstrated that the two engineered probiotics, *EcNT (pTLS)* and *EcNT (pK25)*, were capable of cascading the metabolism of redundant lysine and its metabolites. The cocktail was less sensitive to oxygen *in vitro*, and showed the highest metabolic capacity to the high initial concentration of lysine when the radio of *EcNT (pTLS)*: *EcNT (pK25)* was 1:2. We have also illustrated the artificial microsphere system to effectively solve the problem of spatial positioning in the gut. Our cascade cocktail significantly reduced the concentration of lysine and saccharopine in the plasma of hyperlysinemia-like mice, which is potentially suitable for balancing the concentration of lysine for protein synthesis and the downstream metabolites of lysine metabolism.

## Data availability statement

The original contributions presented in the study are included in the article/Supplementary material, further inquiries can be directed to the corresponding authors.

## Ethics statement

The animal study was approved by Animal protocols were approved by the Ethics committee of the Binzhou Medical University No. 2021049 and all experiments were conducted following the National Guidelines for the Ethical Review of Laboratory Animal Welfare of the People’s Republic of China (GB/T 35892–2018). The study was conducted in accordance with the local legislation and institutional requirements.

## Author contributions

FG: Conceptualization, Data curation, Formal analysis, Funding acquisition, Investigation, Methodology, Project administration, Resources, Software, Supervision, Validation, Visualization, Writing – original draft, Writing – review & editing. MW: Conceptualization, Data curation, Formal analysis, Investigation, Methodology, Resources, Software, Validation, Writing – original draft. PY: Data curation, Formal analysis, Writing – original draft. XL: Supervision, Visualization, Writing – review & editing. XP: Supervision, Visualization, Writing – review & editing. YW: Data curation, Formal analysis, Funding acquisition, Software, Visualization, Writing – original draft, Writing – review & editing. JL: Conceptualization, Data curation, Formal analysis, Funding acquisition, Investigation, Methodology, Project administration, Resources, Software, Supervision, Validation, Visualization, Writing – original draft, Writing – review & editing.
