# Supplementary material for: Engineered probiotic cocktail with two cascade metabolic Escherichia coli for the treatment of hyperlysinemia
Source: Front Microbiol. 2024 May 30;15:1366017. doi: 10.3389/fmicb.2024.1366017 (PMC11169572; doi:10.3389/fmicb.2024.1366017)
Supplement: Supplementary file 1 [file Table_1.DOCX]

# Engineered probiotic cocktail with two cascade metabolic *Escherichia coli* for the treatment of hyperlysinemia

**Table S1 primers list**

| Primer name | Sequence 5`-3` |
| --- | --- |
| pTrc99a-XhoI-F | CTGACACTCGAGTGTTTTGGCGGATGAGAGAAGATTTTCAG |
| pTrc99a-EcorI-R | GAGCTCGAATTCCATGGTCTGTTTCCT |
| SDR-XhoI-R | CGTACTCGAGAGCCACTGTCTTTTCCTTTAGATAGATG |
| SDR-NdeI-F | CGCTCATATGGGAAAGAACGTTTTGTTGCTAG |
| LKR-EcoRI-F | CTAGGAGAATTCTGTTTAACTTTAAGAAGGAGATATACCATGGG |
| LKR-XbaI-R | CATACGTCTAGACAATCTTGAAGATCTTTTAACACGAGCGCAAT |
| LKR-PCR-NdeI-F | CGCTCATATGGCTGCCGTCACATTACATCTAAGAG |
| SDR-NheI-F | CTAGTGGCTAGCTTGTTTAACTTTAAGAAGGAGATATACCATGGGCAG |
| LKR-EcoRI-F | CTAGGAGAATTCTGTTTAACTTTAAGAAGGAGATATACCATGGG |
| pK18-sacB-clone-R | CTTTCGCAAACGCTTGAGTTG |
| pK18-sac-clone-F | TCACATATACCTGCCGTTCA |
| pK18sacB-F | ATGAACATCAAAAAGTTTGCAAAACAAGCAAC |
| pK18sacB-R | TTTTATGTACTGTGTTAGCGGTCTGCTTC |
| lys5-EcoRI-F | ATCGGAGAATTCATGGTTAAAACGACTGAAGTAGTAAGCGAAG |
| lys5-XbaI-R | CGCGGCTCTAGACGATGAAAGGAGGTCTAGGCT  TATAAACCATCATTTTCGATGAAATAATCAATCAACGTAG |
| lys2-XbaI-F | ATGCTCTCTAGAATGACTAACGAAAAGGTCTGGATAG |
| lys2-HindIII-R | ATCGTCAAGCTTTTAAGCTGCTGCGGAGCTTCCACG |

**Table S2 primers list**

| Primer name | Sequence 5`-3` |
| --- | --- |
| pREDCas9-F | CGGATAACGTTCCAAGTGAAGAAGTAGTC |
| pREDCas9-R | CACATATTTGCTTGGCAGAGCCAG |
| ldcC1-gRNA-up | AGGTATAATACTAGTCTTTGAATATGCGCTGGGTGAGTTT  TAGAGCTAGAAATAGCAAGTTAAAATAAGGCTAGTCC |
| ldcC2-gRNA-up | AGGTATAATACTAGTCAATATGCGTATTCAGGATCAGTTTTAGAGC  TAGAAATAGCAAGTTAAAATAAGGCTAGTCC |
| gRNA-down | ACTAGTATTATACCTAGGACTGAGCTAGCTGTCAAG |
| check-sgRNA-F | CGATGTAACCCACTCGTGCAC |
| check-sgRNA-R | AGGGAGAAAGGCGGACAGGTA |
| ldcC2-down-R | CGGCGATGACAAGCCTTACTG |
| ldcC2-if-donw-F | ATGAACATCATTGCCATTATGGGACCGCCGCGTACGAGTCCTAAAAATGG |
| ldcC2-up-R | CGGTCCCATAATGGCAATGATGTTC |
| ldcC2-up-F | GCGGTCAGTCTGAAGCCATTG |
| ldcC2-seq-F | CGTAATCGGCAAATGTTCTGAAAGG |
| ldcC1-up-R | GACGGTAACTTCCCGAAAGTTTATG |
| ldcC1-up-F | CCCATGTGATTCAATATTGCAATAACGTTCATA |
| ldcC1-seq-F | GCCAGAGCCACTCAATGGATAAC |
| gRNA-ldcC1 | CTTTGAATATGCGCTGGGTG |
| ldcC1-if-down-F | GAACGTTATTGCAATATTGAATCACATGGGGTGC  ATACCGTCAGGCTGATGG |
| ldcC1-down-F | GTGCATACCGTCAGGCTGATGG |
| ldcC1-down-R | TCGATGATCGTTCTCGTCGTAGAGCTC |
